# Supplementary material for: The spatial transcriptomic landscape of non-small cell lung cancer brain metastasis
Source: Nat Commun. 2022 Oct 10;13:5983. doi: 10.1038/s41467-022-33365-y (PMC9551067; doi:10.1038/s41467-022-33365-y)
Supplement: Supplementary file 1 — Supplementary Information [file 41467_2022_33365_MOESM1_ESM.pdf]

Supplementary Information

The spatial transcriptomic landscape of non-small cell lung cancer brain metastasis

Qi Zhang, Rober Abdo, Cristiana Iosef, Tomonori Kaneko, Matthew Cecchini,
Victor K Han and Shawn SC Li

Supplementary Information contains

10 Supplementary Figures

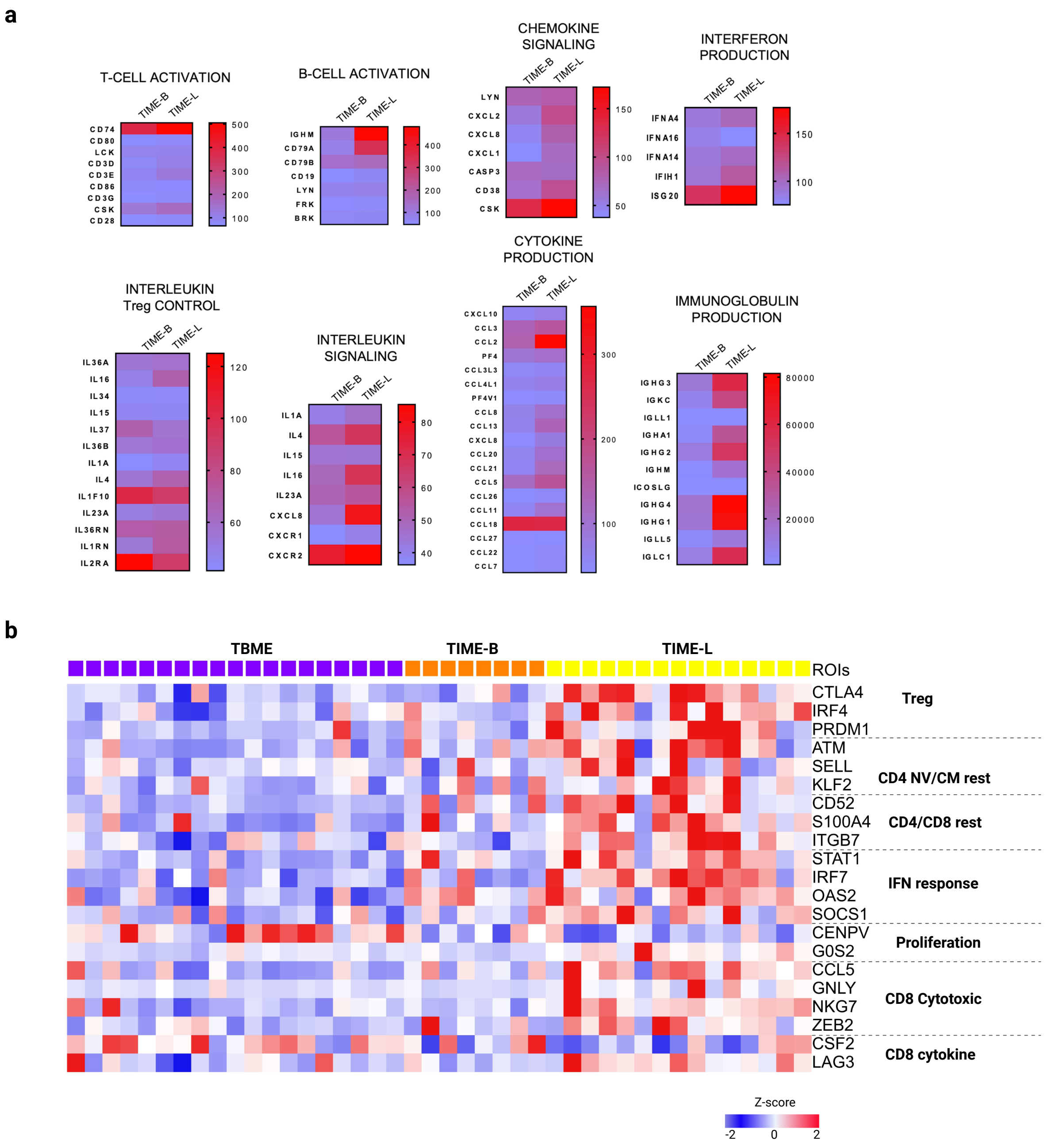

Supplementary Figure 1 (Supportive Data to Main Fig. 2). The brain tumor microenvironment is immunosuppressed compared to the primary lung tumor. (a) Heatmaps of gene expression between the TIME-L and TIME-B for selective markers of T/B cell activation, immunogloblins, cytokines and chemokines. (b) A heatmap of statistically significant genes of T cell modules[1] between TIME-L, TIME-B and TBME. Source data are provided as a Source Data file.

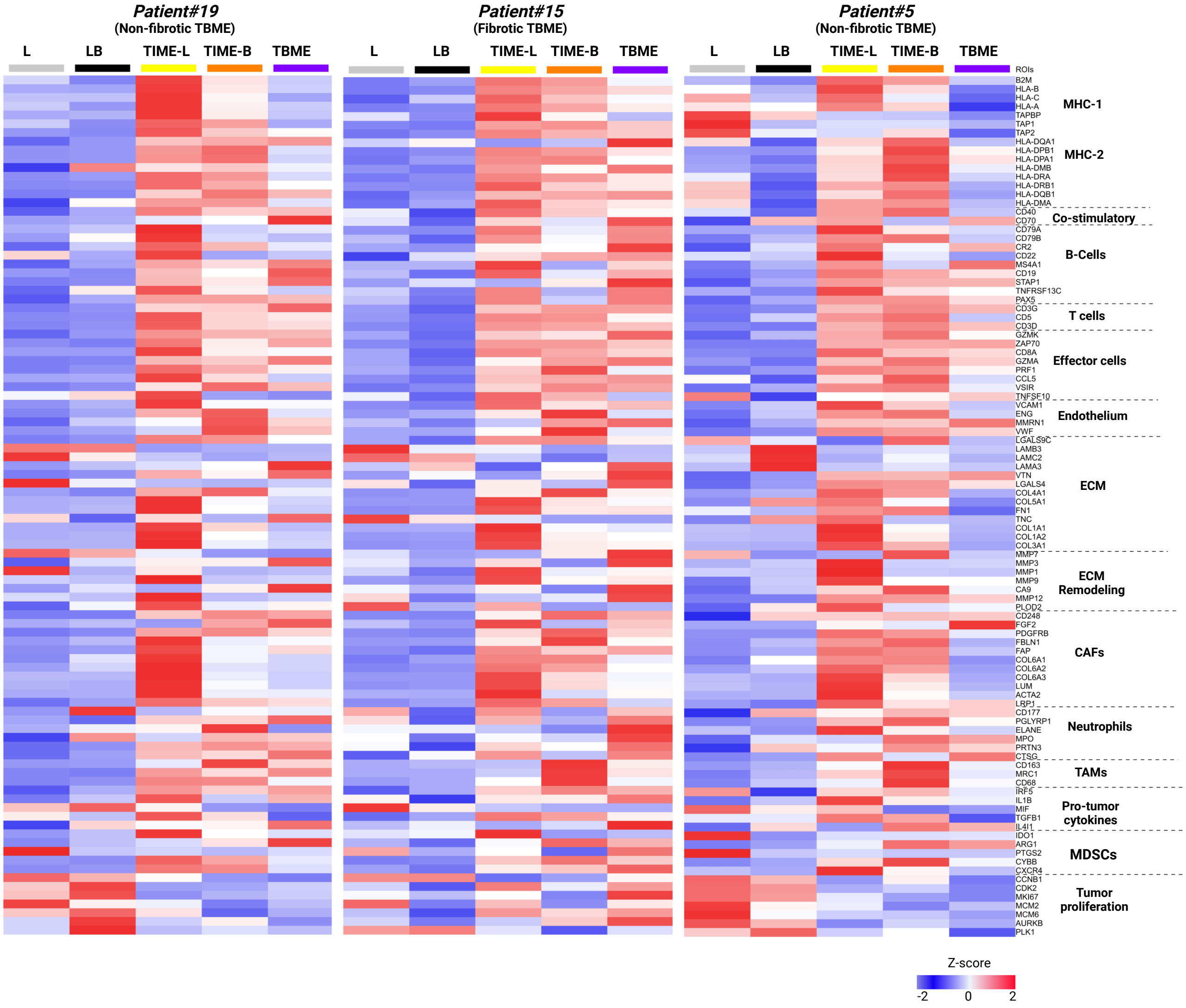

Supplementary Figure 2 (Supportive Data to Main Fig. 3) Intra-patient heterogeneity in the tumor and the TME between the primary tumor and brain metastasis. A heatmap of functional gene signatures (Fges) to show disparities between the cellular and extracellular components in the different regions of the primary and metastasized tumors for the same patient. Data from three patients with matched tissues are shown. Source data are provided as a Source Data file.

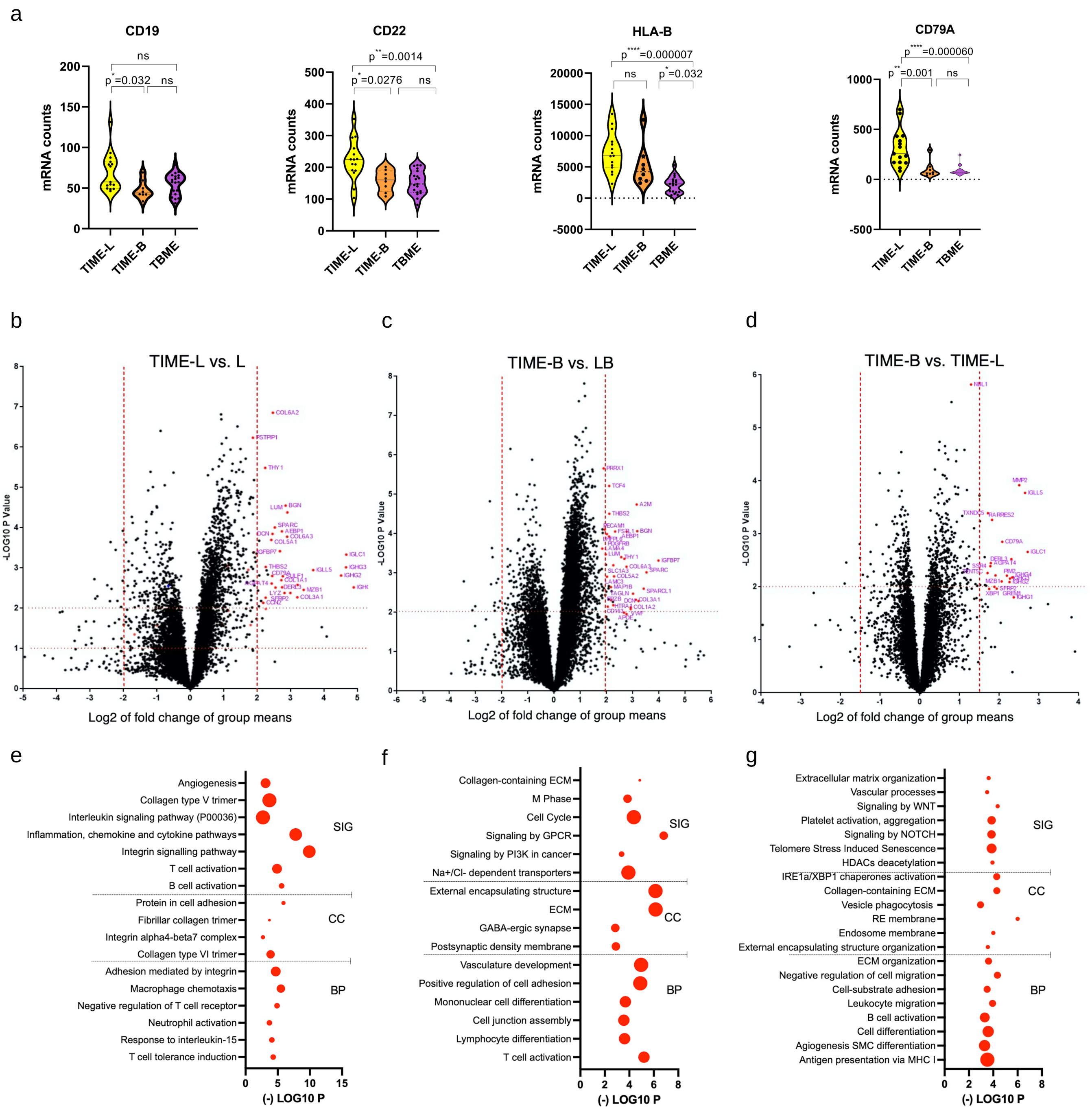

Supplementary Figure 3 (Supportive Data to Main Fig. 3) Differential gene expression and the associated changes in cellular function between the lung and brain TME. (a) Expression of CD19, CD22, HLA-B or CD79A was significantly increased in the TIME-L (n=15) compared to TIME-B (n=8) or TBME (n=19) . P values shown were based on Kruskal Wallis test followed by Dunn test for pairwise comparisons. The dashed and solid lines within the plots indicate upper & lower quartiles and medians respectively. (b-d) Volcano plots comparing gene expression in TIME-L vs. L,TIME-B vs. LB and TIME-B vs. TIME-L. The x-axis indicates fold changes in gene expression between groups in Log2 scale and the Y-axis shows the corresponding P value in -Log10 scale. (e-g) Gene Oncology annotation of the differentially expressed genes (DEGs) identified in the Volcano plots. The size of the bubble is proportional to the fold-change (4-30 folds) whereas the position of the bubble on the x-axis corresponds to the P value (in -Log10 scale). SIG: Signaling process; CC, Cellular Components; BP: Biological Process. p-values in (b-g) were obtained from Mann-Whiteney test (two-sided). Source data are provided as a Source Data file.

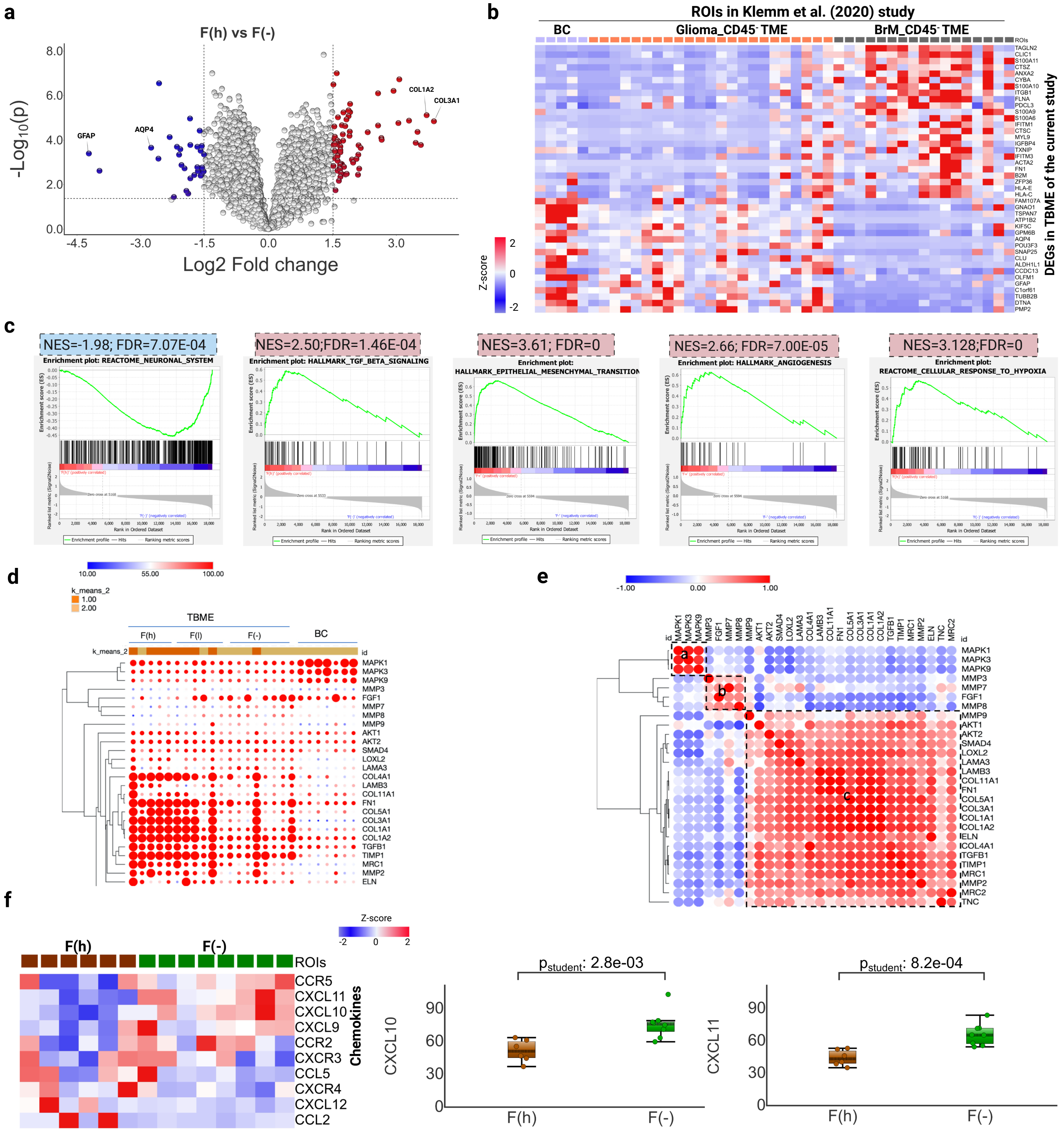

Supplementary Figure 4 (Supportive Data to Main Fig. 4). Distinct gene expression patterns between the fibrous and non-fibrous TBME. (a) Volcano plot showing differentially expressed genes in the F(h) vs F(-) TBME. p-values were obtained from the student t-test (two-sided). (b) heatmap of DEGs in the TBME of the current study validated in CD45^{neg} compartments of BrM in Klemm et al.(2020) study[2]. (c) GSEA plots of ranked gene expression in the neuronal and TGFβ signaling pathway, angiogenesis, EMT , and response to hypoxia . FDR values were obtained from a permutation test. (d) Heatmap depicts clustering of ECM genes in TBME and BC along with their predicted inter-relationship based on tstochastic neighbor embedding (tSNE, T1/2). The bubble size and color were proportionate to the relative level of gene expression in each row. (e) Heatmap from clustering of the same genes using the ‘Pearson minus one’ correlation algorithm. (f) heatmap of chemokines across non-fibrotic F(-) (n=8) and highly fibrotic F(h) (n=6) TBMEs with box sample plots displaying higher expression of CXCL10 and CXCL11 in F(-). p-values were obtained from the student t-test (two-sided). The limits of the box plots represent upper & lower quartiles, whereas the dashed lines indicate medians. Erros bars represent SD. Source data are provided as a Source Data file.

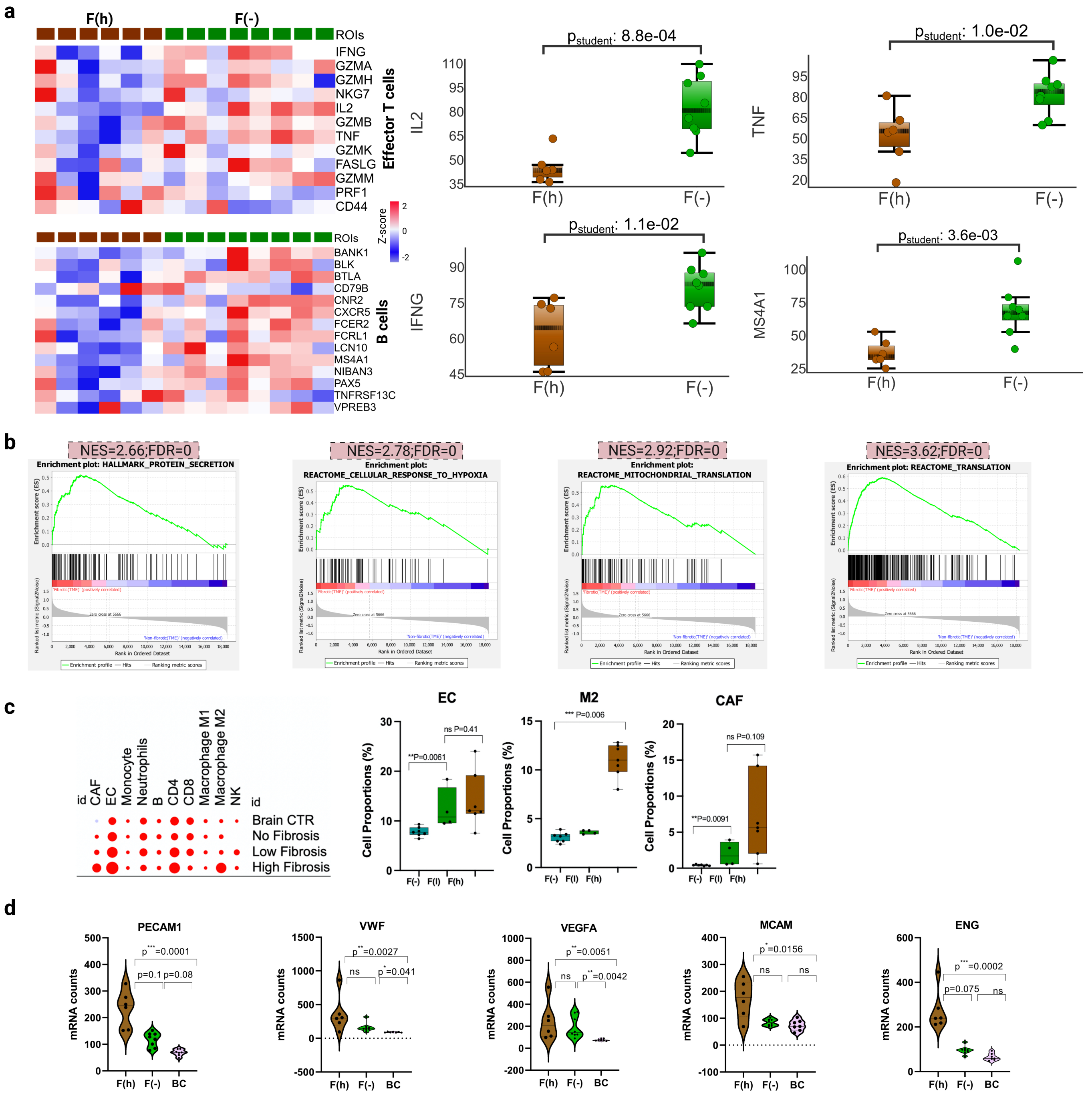

Supplementary Figure 5 (Supportive Data to Main Fig. 4). The cellular composition and function of the brain tumor microenvironment are defined by fibrosis. (a) heatmap of signature genes of effector T cell and B cells along with box plots showing the significantly suppressed expression of IL-2, IFNG, TNF, and MS4A1 in F(h) (n=6) relative to F(-) (n=8). P values were obtained by student t- test (two-sided). The dotted lines within the boxes indicate medians, whereas the box limits define the upper and lower quartiles. Erros bars represent SD. (b) GSEA plots highlighting the enrichment of pathways associated with protein secretion, response to hypoxia, translation, and mitochondria activities in the LB hosted by fibrotic TME. FDR values were obtained from a permutation test. (c) Cell deconvolution by CIBERSORTx identified differences in immune and stromal cell populations in the TBME associated with the different fibrosis state. Scatter plots highlight the differences in endothelial cells (EC), cancer-associated fibroblasts (CAF) and M2 macrophages between the F(h) (n=6), F(l) (n=5) and F(-) (n=8) TBMEs. p-values were obtained from Mann-Whiteney test (two-sided). Erros bars represent SD. (d) Violin plots of mRNA levels for selected endothelial or angiogenesis markers among F(h) TBME (n=6), F(-) TBME (n=8), and BC (n=7).P values shown were based on Kruskal-Wallis test followed by Dunn test for pairwise comparisons. Within violin plots in (d) and box plots in (c), the dashed lines indicate upper & lower quartiles, whereas the solid lines represent medians. Source data are provided as a Source Data file.

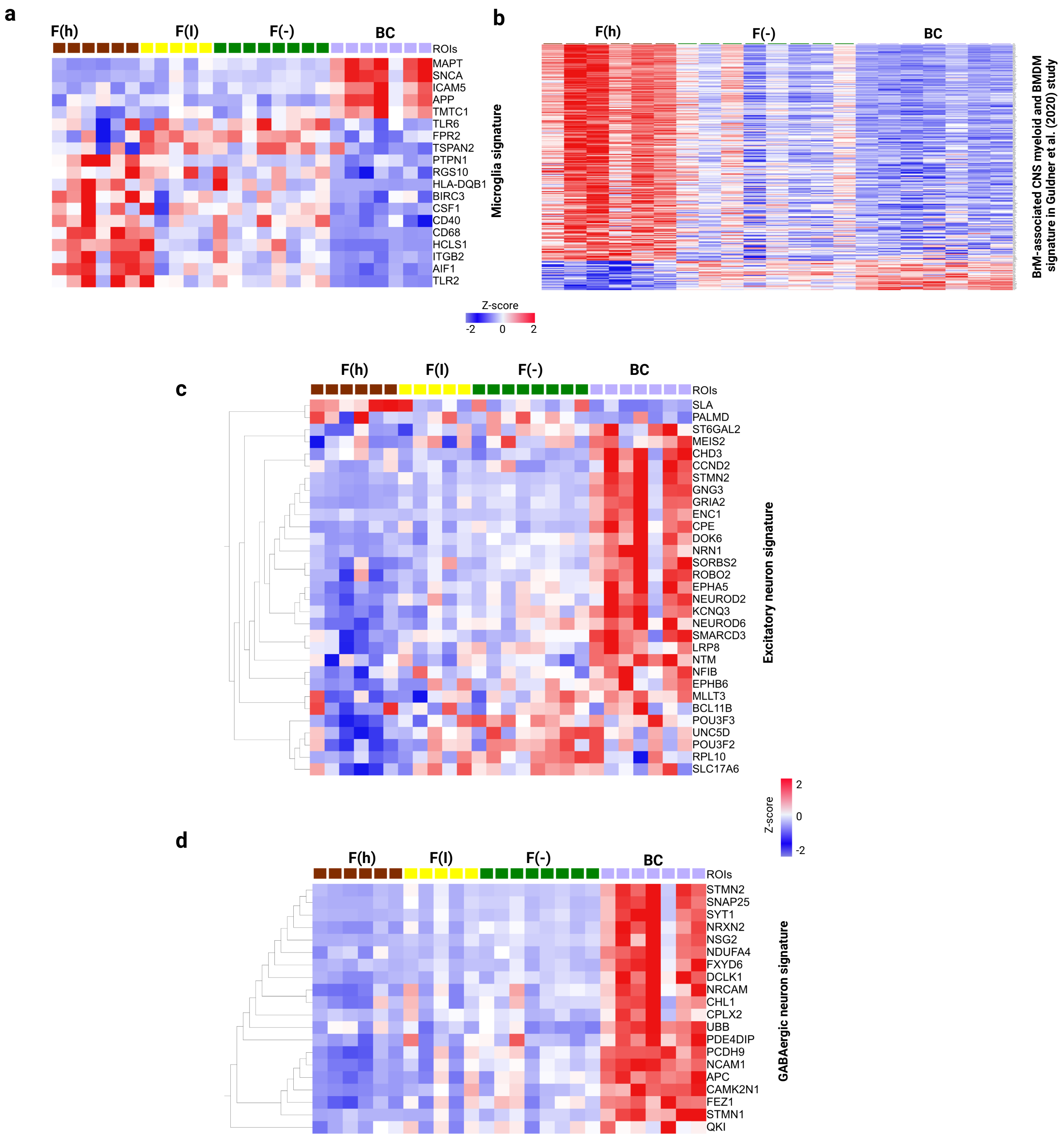

Supplementary Figure 6 (Supportive Data to Main Fig. 4 and Fig. 5). Microglia and neuronal functions are compromised in the tumor brain microenvironment. Significantly differentially expressed signature genes between the TBME and BC for (a) microglia, (b) BrM-associated CNS-myeloid and BMDMs[3]. Supervised hierarchical clustering of the significantly expressed genes between TBME and BC for (c) excitatory neurons and (d) GABAergic neurons in the TBME and brain control tissues. Heatmaps colored according to Z-score. Source data are provided as a Source Data file.

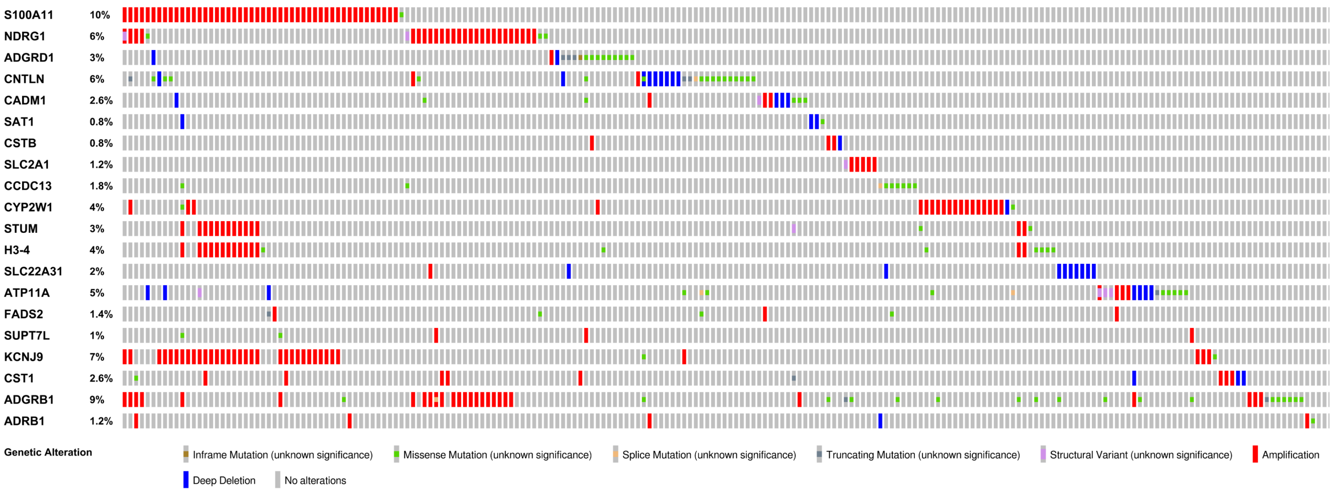

Supplementary Figure 7 (Supportive Data to Main Fig. 8). The metastasis signature genes are altered in LAUD. Shown are rates of alteration for the metastasis signature genes in the TCGA LUAD cohort. The graph was generated using the cBioPortal for Cancer Genomics (https://cbioportal.org).

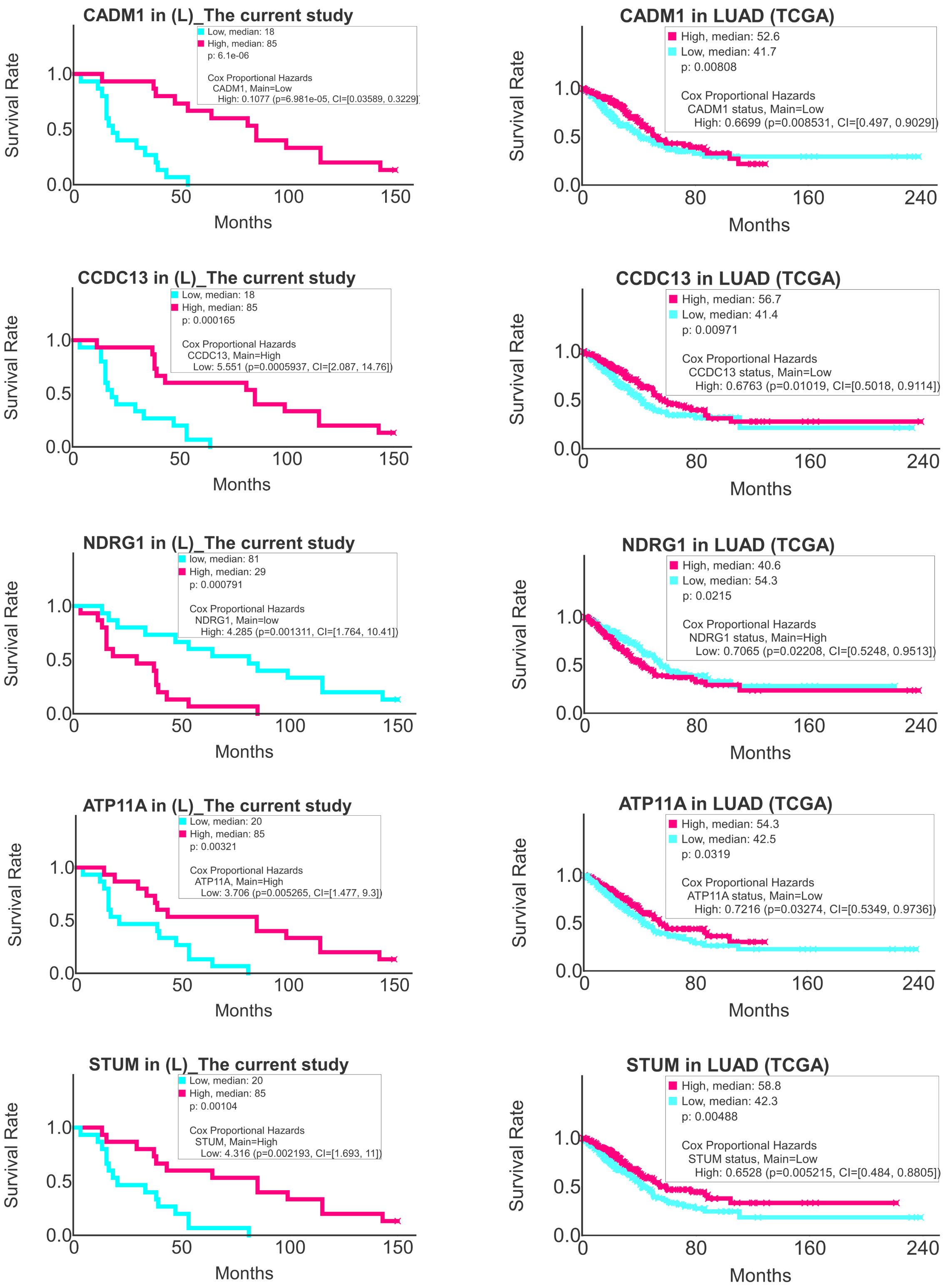

Supplementary Figure 8 (Supportive Data to Main Fig. 8). Signature genes of lung cancer metastasis predicted patient outcome. Left panels, current cohort (n=30); right panels, TCGA LUAD cohort (n=501). P values were based on Logrank test.The analyses were accompanied by cox proportional hazard ratios with the confidence intervals and p-values. Source data are provided as a Source Data file.

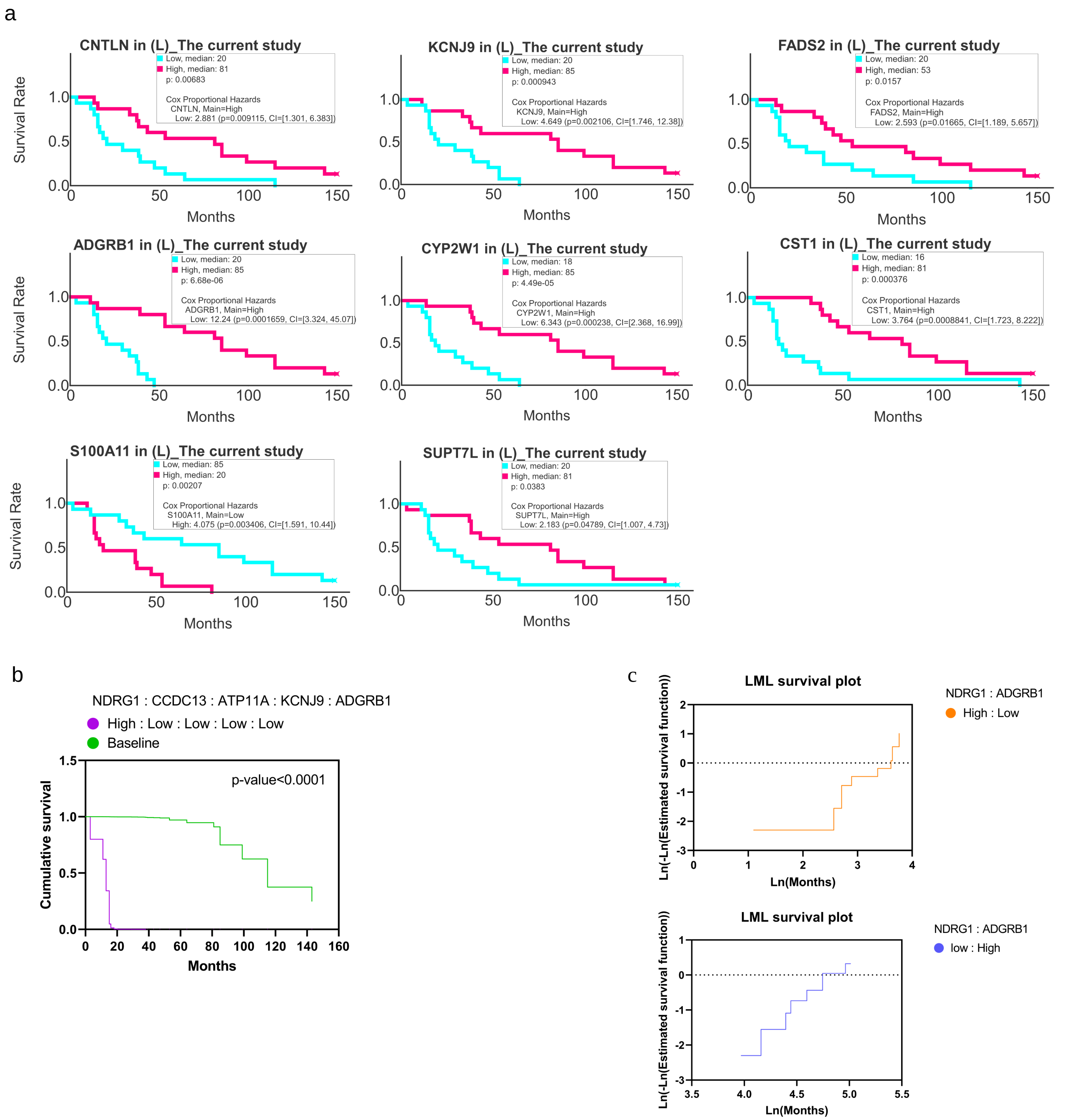

Supplementary Figure 9 (Supportive Data to Main Fig. 8). Signature genes of lung cancer metastasis predicted patient outcome. (a) Metastasis signature genes correlated with survival in the current study cohort (n=30). P values were based on Logrank test for Kaplan Meier analysis and cox proportional hazard ratio was performed on individual genes with p-values and confidence intervals displayed. (b) Covariates-Cox regression model with a set of 5 significantly interrelated metastasis genes. The median survival is severely compromised for patients exhibiting the expression pattern of the 5 genes as shown above the graph. (c) Cumulative hazard function plot using the Nelson-Aalen estimator method for the two most significant genes (NDRG1 &ADGRB1) in the metastasis signature model. The high/low expression of NDRG1/ADGRB1 was associated with a risker hazard ratio. Source data are provided as a Source Data file.

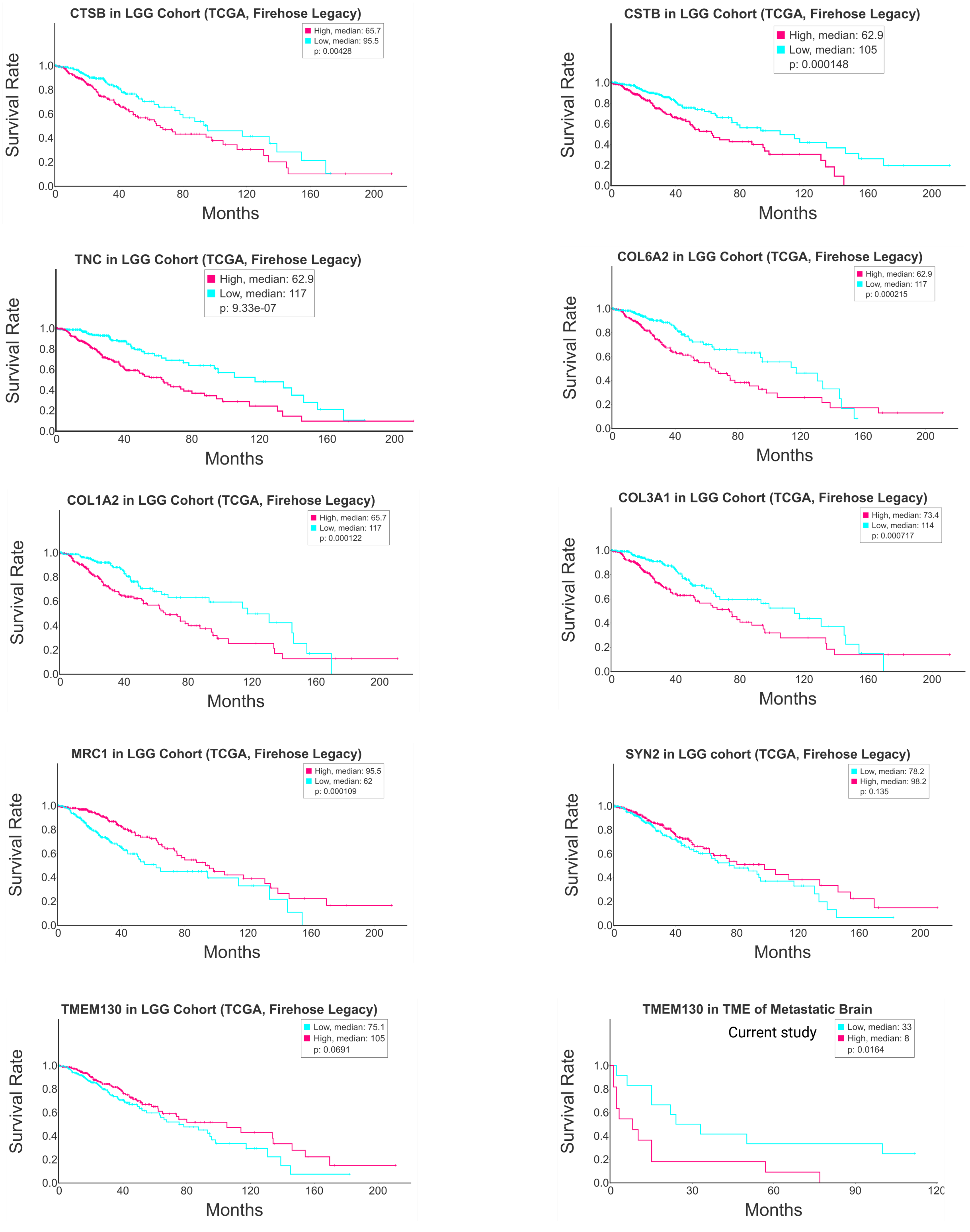

Supplementary Figure 10 (Supportive Data to Main Fig. 8). Identification of biomarkers for predicting survival of brain cancer patients. The TCGA LGG cohort (n=515) was used for prediction except for TMEM130 where both the LGG and the current cohort were used. P values were based on Logrank test.

Supplementary References

[1] P. A. Szabo *et al.*, “Single-cell transcriptomics of human T cells reveals tissue and activation signatures in health and disease,” *Nat. Commun.*, vol. 10, no. 1, Art. no. 1, Oct. 2019, doi: 10.1038/s41467-019-12464-3.

[2] F. Klemm *et al.*, “Interrogation of the Microenvironmental Landscape in Brain Tumors Reveals Disease-Specific Alterations of Immune Cells,” *Cell*, vol. 181, no. 7, pp. 1643-1660.e17, Jun. 2020, doi: 10.1016/j.cell.2020.05.007.

[3] I. H. Guldner *et al.*, “CNS-Native Myeloid Cells Drive Immune Suppression in the Brain Metastatic Niche through Cxcl10,” *Cell*, vol. 183, no. 5, pp. 1234-1248.e25, Nov. 2020, doi: 10.1016/j.cell.2020.09.064.
