# Supplementary material for: The spatial transcriptomic landscape of non-small cell lung cancer brain metastasis
Source: Nat Commun. 2022 Oct 10;13:5983. doi: 10.1038/s41467-022-33365-y (PMC9551067; doi:10.1038/s41467-022-33365-y)
Supplement: Supplementary file 3 — Description of Additional Supplementary Files [file 41467_2022_33365_MOESM3_ESM.pdf]

Description of Supplementary Data files

Supplementary Data 1. Demographics and Pathological Information of the Study Patient Cohort. The age of NSCLC diagnosis among the patients ranges from 46 to 97 years old. Adenocarcinoma is the predominant histological type of primary NSCLCs and BrMs across the patients. The majority of primary NSCLCs and BrMs were treated with radiation and chemotherapies.

Supplementary Data 2. Fibrosis Scores for ROIs in TBME ROIs. Based on staining with Masson Trichrome, nineteen TBME ROIs from nineteen patients were initially classified into negative stained ROIs (eight patients) and positive stained ROIs (eleven patients). The Masson Trichrome-positive stained ROIs were further quantified using a positive pixel method by QuPath software and subsequently sub-classified into a highly fibrotic group (6 patients) and an intermediately fibrotic group (5 patients) based on a median cut-off.

Supplementary Data 3. Differentially expressed genes (DEGs) between histologically or spatially varied ROIs. Four tables respectively reveal the DEGs between (TBME) ROIs and (BC) ROIs, (TIME-B) ROIs and (LB) ROIs, (TIME-L) ROIs and (L) ROIs, and between the ROIs of highly fibrotic (F(h)) TBME and ROIs of non-fibrotic (F(-)) TBME. p-values were obtained from the student t-test (two-sided).

Supplementary Data 4. Differentially expressed genes (DEGs) between ROIs correspond to the status of the metastatic intervals to the brain (Fast metastasis vs. slow metastasis). (L) ROIs were grouped into the fast metastasis group (The first tertile, ten patients) and the slow metastasis group (The third tertile, ten patients), dismissing the second tertile (10 patients) to avoid cases with adjacent time intervals to the brain. Accordingly, four tables of DEGs were executed between fast metastasis (L) ROIs and slow metastasis (L) ROIs and between corresponding fast metastasis (LB) ROIs and slow metastasis (LB) ROIs. The third and fourth tables illustrate the DEGs between ROIs of TME in the brain hosting fast metastasis tumors and ROIs of TME in the brain hosting slow metastasis tumors and between (TIME-L) ROIs hosting fast metastasis and (TIME-L) ROIs hosting slow metastasis. p-values were obtained from the student t-test (two-sided).
