# Supplementary material for: The spatial transcriptomic landscape of non-small cell lung cancer brain metastasis
Source: Nat Commun. 2022 Oct 10;13:5983. doi: 10.1038/s41467-022-33365-y (PMC9551067; doi:10.1038/s41467-022-33365-y)
Supplement: Supplementary file 8 — Reporting Summary [file 41467_2022_33365_MOESM8_ESM.pdf]

nature portfolio

Corresponding author(s): Shawn Li, Qi Zhang

Last updated by author(s): Sep 9, 2022

Reporting Summary

Nature Portfolio wishes to improve the reproducibility of the work that we publish. This form provides structure for consistency and transparency in reporting. For further information on Nature Portfolio policies, see our Editorial Policies and the Editorial Policy Checklist.

Statistics

For all statistical analyses, confirm that the following items are present in the figure legend, table legend, main text, or Methods section.

n/a	Confirmed	
☐	☒	The exact sample size (*n*) for each experimental group/condition, given as a discrete number and unit of measurement
☐	☒	A statement on whether measurements were taken from distinct samples or whether the same sample was measured repeatedly
☐	☒	The statistical test(s) used AND whether they are one- or two-sided<br>*Only common tests should be described solely by name; describe more complex techniques in the Methods section.*
☐	☒	A description of all covariates tested
☐	☒	A description of any assumptions or corrections, such as tests of normality and adjustment for multiple comparisons
☐	☒	A full description of the statistical parameters including central tendency (e.g. means) or other basic estimates (e.g. regression coefficient) AND variation (e.g. standard deviation) or associated estimates of uncertainty (e.g. confidence intervals)
☐	☒	For null hypothesis testing, the test statistic (e.g. *F*, *t*, *r*) with confidence intervals, effect sizes, degrees of freedom and *P* value noted<br>*Give P values as exact values whenever suitable.*
☒	☐	For Bayesian analysis, information on the choice of priors and Markov chain Monte Carlo settings
☒	☐	For hierarchical and complex designs, identification of the appropriate level for tests and full reporting of outcomes
☒	☐	Estimates of effect sizes (e.g. Cohen's *d*, Pearson's *r*), indicating how they were calculated

Our web collection on statistics for biologists contains articles on many of the points above.

Software and code

Policy information about availability of computer code

Data collection	GeoMx® DSP data analysis software v2.1 from NanoString for normalizing the RNA counts. DND pipeline was used, Nanostring manual ID: MAN-10118-01
Data analysis	Qlucore Omics Explorer 3.8.2<br>GSEA v4.2.1 software<br>Cytoscape software 3.9.0<br>GraphPad v9.3.1<br>STRING v11.5

For manuscripts utilizing custom algorithms or software that are central to the research but not yet described in published literature, software must be made available to editors and reviewers. We strongly encourage code deposition in a community repository (e.g. GitHub). See the Nature Portfolio guidelines for submitting code & software for further information.

Data

Policy information about availability of data

All manuscripts must include a data availability statement. This statement should provide the following information, where applicable:

- Accession codes, unique identifiers, or web links for publicly available datasets
- A description of any restrictions on data availability
- For clinical datasets or third party data, please ensure that the statement adheres to our policy

The raw and processed RNA sequencing data generated in this study is available in Gene Expression Omnibus (GEO) with the assigned provisional Series accession number GSE200563 (https://www.ncbi.nlm.nih.gov/geo/query/acc.cgi?acc=GSE200563) . The LUAD cohort (TCGA, PanCancer Atlas) and the LGG (TCGA, Firehose Legacy) were obtained from CbioPortal.org. The FDA approved therapeutic targets were taken from the DrugBank database (drugbank.ca) . The gene markers for astrocytes and NK cells were obtained from the PanglaoDB database (https://panglaodb.se/) . Source data are provided with this paper. The remaining data are available within the Article, Supplementary Information or Source Data file.

Human research participants

Policy information about studies involving human research participants and Sex and Gender in Research.

Reporting on sex and gender	Our study is a retrospective study with samples collected in the past 15 years; therefore, sex/gender representation is reflected in whatever samples we accrued. Our analysis did not focus on identifying sex/gender-specific differences. Instead, we intended to identify the molecular signature of primary NSCLC that has a propensity to metastasize to the brain and the molecular plasticity of the tumor microenvironment in the brain regardless of sex/gender variables. The findings of the study are applicable to an NSCLC-BrM patient regardless of sex/gender identity.
Population characteristics	Detailed patient information is listed in Supplementary Data 1
Recruitment	There was no recruitment biases to impact the results of our analyses.
Ethics oversight	The Western University Research Ethics Board

Note that full information on the approval of the study protocol must also be provided in the manuscript.

Field-specific reporting

Please select the one below that is the best fit for your research. If you are not sure, read the appropriate sections before making your selection.

☒ Life sciences ☐ Behavioural & social sciences ☐ Ecological, evolutionary & environmental sciences

For a reference copy of the document with all sections, see nature.com/documents/nr-reporting-summary-flat.pdf

Life sciences study design

All studies must disclose on these points even when the disclosure is negative.

Sample size	Case search from the Department of Pathology at London Health Sciences Centre (January 2007 to January 2018) identified 293 cases of surgically resected brain metastasis with lung origin. Among these cases, we selected 44 cases with patient-paired primary lung cancer and brain metastasis, where adequate material was available for analysis.
Data exclusions	Cases with extensive necrosis, hemorrhage, or insufficient tissue material are excluded. The cases with the latter features were not included for RNSseq since they hinder the quality and accuracy of RNA counts.
Replication	The spatial RNA sequencing was performed in one experimental run. The Masson Trichrome staining was successfully conducted on TMAs in one experiment run.
Randomization	The NSCLC patients with matched brain metastases along with patients without tumor pathology were selected in the study and its design is not based on randomization
Blinding	The study intended to investigate the disparities between the primary tumors and metastatic tumor and difference in brain landscapes between metastatic brain and non-tumor brain. The investigators were not blinded to allocation during experiments and outcome assessment, so that enough samples were obtained for each group.

Reporting for specific materials, systems and methods

We require information from authors about some types of materials, experimental systems and methods used in many studies. Here, indicate whether each material, system or method listed is relevant to your study. If you are not sure if a list item applies to your research, read the appropriate section before selecting a response.

Materials & experimental systems

n/a	Involved in the study
☐	☒ Antibodies
☒	☐ Eukaryotic cell lines
☒	☐ Palaeontology and archaeology
☒	☐ Animals and other organisms
☒	☐ Clinical data
☒	☐ Dual use research of concern

Methods

n/a	Involved in the study
☒	☐ ChIP-seq
☒	☐ Flow cytometry
☒	☐ MRI-based neuroimaging

Antibodies

Antibodies used	GFAP (Invitrogen, 53-9892-82), CD45 (Biolegend, 121302310), and PanCK (Novus, NBP2-33200AF647) and Syto83 (ThermoFisher, S11364).
Validation	Used antibodies were titrated or used as recommended by the manufacturer. All the antibodies are validated for use in flow cytometry. Species validation and antibody data are available on the manufacturer's website. All used antibodies are commercially available.
